# The persistent chasm between PrEP awareness and uptake: characterizing the biomedical HIV prevention continuum in a nationwide cohort of transgender women in the United States and Puerto Rico

**DOI:** 10.1002/jia2.70070

**Published:** 2025-12-18

**Authors:** Erin E. Cooney, Tonia C. Poteat, Meg Stevenson, Asa E. Radix, Annick Borquez, Keri N. Althoff, Sabriya Linton, Ceza Pontes, Chris Beyrer, Arianna Lint, Marissa Miller, Carter Brown, Andrew J. Wawrzyniak, Carolyn A. Brown, Leigh Ragone, Vani Vannappagari, Adrienne Guignard, Sari L. Reisner, Andrea L. Wirtz, ENCORE Study Group

**Affiliations:** ^1^ Department of International Health Johns Hopkins Bloomberg School of Public Health Baltimore Maryland USA; ^2^ Center for Public Health and Human Rights Department of Epidemiology Johns Hopkins Bloomberg School of Public Health Baltimore Maryland USA; ^3^ Duke University School of Nursing Durham North Carolina USA; ^4^ Callen‐Lorde Community Health Center New York New York USA; ^5^ Department of Epidemiology Mailman School of Public Health Columbia University New York New York USA; ^6^ Division of Infectious Diseases & Global Public Health UC San Diego La Jolla California USA; ^7^ Department of Epidemiology Johns Hopkins Bloomberg School of Public Health Baltimore Maryland USA; ^8^ Department of Mental Health Johns Hopkins Bloomberg School of Public Health Baltimore Maryland USA; ^9^ Duke Global Health Institute Duke University Durham North Carolina USA; ^10^ Arianna's Center Fort Lauderdale Florida USA; ^11^ TransSolutions Research & Resource Center Indianapolis Indiana USA; ^12^ Black Trans Advocacy Coalition Carrollton Texas USA; ^13^ Department of Psychiatry and Behavioral Sciences University of Miami Miller School of Medicine Miami Florida USA; ^14^ ViiV Healthcare Durham North Carolina USA; ^15^ ViiV Healthcare Wavre Belgium; ^16^ Department of Epidemiology University of Michigan School of Public Health Ann Arbor Michigan USA; ^17^ The Fenway Institute Boston Massachusetts USA

**Keywords:** transgender women, HIV prevention, pre‐exposure prophylaxis, uptake, adherence

## Abstract

**Introduction:**

Transgender (trans) women are disproportionately impacted by HIV, yet data on the biomedical HIV PrEP continuum (HIVPC) among trans women are limited. We characterized the HIVPC among a large, nationwide cohort of trans women in the United States and Puerto Rico by pre‐exposure prophylaxis (PrEP) modality (daily oral and long‐acting injectable, LAI) and identified correlates of uptake and non‐adherence.

**Methods:**

From April 2023 to December 2024, we enrolled English and Spanish‐speaking adult trans women (age 18 years or older) not living with HIV (laboratory‐confirmed via fourth‐generation HIV‐1/2 antigen/antibody testing) and residing in the United States and Puerto Rico into the cohort. PrEP data were collected via self‐administered surveys. We characterized the HIVPC using descriptive statistics and assessed for differences in proportions for each step of the HIVPC by modality. Modified Poisson regression models estimated adjusted prevalence ratios (aPR) and 95% confidence intervals (95% CI) for correlates of HIVPC step (e.g. awareness to uptake).

**Results:**

We enrolled 2504 participants, 1636 (65%) of whom may have benefitted from PrEP based on self‐reported sexual history and/or needle sharing in the prior 6 months at baseline. Forty‐two percent were 18–29 years old, 18% identified as Hispanic and/or Latina/x/e and 13% identified as Black (inclusive of multiracial participants). Among participants who may have benefitted from PrEP, 92% (*n* = 1495) had ever heard of PrEP, 36% (*n* = 591) had ever used PrEP, 27% (*n* = 441) had recently used PrEP (past 6 months) and 20% (*n* = 330) were adherent. The largest proportional difference in HIVPC step was from awareness to uptake (60% of PrEP‐aware participants had never used PrEP). This difference was significantly greater for LAI PrEP (96% of LAI PrEP‐aware participants had never used LAI). Correlates of PrEP uptake included high perceived HIV acquisition risk (aPR = 2.08, 95% CI = 1.59−2.72; ref = no perceived risk), current use of exogenous oestrogen and/or anti‐androgens (aPR = 1.47 95% CI = 1.21−1.79), and receipt of health services at an LGBTQ+ clinic (aPR = 1.34, 95% CI = 1.16−1.55). Correlates of non‐adherence among PrEP users included being a non‐U.S. citizen (aPR: 2.41, 95% CI = 1.44−4.05) and recent food insecurity (aPR: 1.47, 95% CI = 1.04−2.06).

**Conclusions:**

Interventions to improve HIVPC outcomes—especially PrEP uptake—are needed to optimize HIV PrEP among trans women. PrEP interventions may need to include individually tailored, integrated programming to address risk perception, nutrition, gender‐affirming care and comprehensive health, social, and legal needs.

## 1 INTRODUCTION

Transgender (trans) women have the highest HIV prevalence of all priority populations—defined in the U.S. National HIV/AIDS Strategy as populations identified in national‐level data as being disproportionately affected by HIV—with an estimated 14–19% of trans women living with HIV [1, 2]. There are notable inequities by race and ethnicity, with as many as 40–50% of Black trans women living with HIV and as many as 33% of Latina trans women living with HIV [1]. There is an extensive body of research documenting the barriers to healthcare that trans women face, including multiple intersecting socio‐economic and structural barriers to care [3−6]. These barriers impede engagement in HIV prevention services specifically, highlighting the necessity of addressing them to achieve optimal engagement in the HIV PrEP continuum (HIVPC) [7]. The HIVPC is a heuristic framework which emphasizes HIV prevention as a spectrum of engagement that requires multiple steps to optimally mitigate HIV acquisition risk, including pre‐exposure prophylaxis (PrEP) awareness, uptake and adherence [8]. While the prevention continuum includes prevention strategies beyond PrEP (e.g. condoms), the HIVPC emphasizes PrEP as the most efficacious prevention strategy among individuals at risk for HIV acquisition.

Although national HIVPC data among trans women are limited, prior studies in several U.S. metropolitan areas have produced estimates [9−12]. These data suggest trans women have high levels of oral PrEP awareness, varying from 79% in a 2016 study in San Francisco [11] up to 92% in a 2019–2020 National HIV Behavioral Surveillance study in seven cities [12]. This research has reported suboptimal PrEP uptake among those who may benefit, varying from 15% in the 2016 study in San Francisco [11] to 38% in a 2018–2020 multi‐city study in the eastern and southern United States [10]. These studies estimate that just 10–13% of trans women who could benefit from PrEP are adherent PrEP users [10, 11]. Thus, evidence from sub‐national samples over the last decade consistently finds high awareness and willingness to use PrEP among trans women but low levels of uptake and adherence [9]. Despite the availability of these data for some large metropolitan areas, we were not able to identify extant HIVPC data from national samples of trans women. Further, published findings are almost exclusively from studies conducted prior to the availability of long‐acting injectable (LAI) PrEP, which was approved by the U.S. Food and Drug Administration in December 2021.

Given this lack of recent, national HIVPC data, we sought to describe levels of awareness, uptake and adherence to PrEP (daily oral and LAI regimens) among trans women in the United States. We aimed to describe the socio‐demographics and experiences of LAI PrEP users in our sample. Further, we sought to identify characteristics associated with a step in the HIVPC (i.e. PrEP uptake among those aware of PrEP) to inform interventions designed to improve uptake and adherence among trans women.

## 2 METHODS

### 2.1 Study population

We used baseline data from ENCORE, which is a large, epidemiological cohort of trans women who are not living with HIV and reside in the United States and Puerto Rico. Participants were eligible to participate if they met the following inclusion criteria: identify as a trans woman or along the trans‐feminine spectrum, speak English and/or Spanish and reside in the United States or Puerto Rico. Participants were excluded from the study if they were determined to be living with HIV at baseline, had previously enrolled in the study or were unable to consent to participate in the study. The primary aims of ENCORE are to evaluate HIV incidence and to investigate associated syndemic conditions (e.g. substance use, violence) among this population [13]. Enrolment opened in April 2023 and ended in December 2024, with *N* = 2504 trans women enrolled. ENCORE uses a novel, hub‐supported digital cohort model, which allows for study components to be completed remotely, enabling nationwide enrolment, while leveraging community‐based hubs in 10 Ending the HIV Epidemic priority jurisdictions [14] to facilitate inclusion of the most marginalized trans women, who may not have reliable access to digital devices required to complete the survey or a consistent place where they can receive HIV test kits by mail [15]. Participation is available in English and Spanish. Procedures to prevent fraudulent enrolments include two‐factor authentication prior to enrolment, address verification, and automated and manual reviews of registration data to identify duplicate enrolments and suspicious activity. Additional ENCORE procedures are available in the published protocol [13].

### 2.2 Measures

PrEP outcomes were self‐reported during the baseline survey. Participants were shown introductory text to describe PrEP prior to answering PrEP questions. Text read: “PrEP is when people who do not have HIV take anti‐HIV medications to prevent getting HIV. PrEP can be in the form of a pill taken daily (often known as Truvada or Descovy) or an injection taken once every two months (often known as Apretude or Cabotegravir).” Survey measures included awareness (i.e. “Have you ever heard of ‘PrEP’ (pre‐exposure prophylaxis) for the prevention of HIV infection in people who are HIV‐negative?”) and uptake and current use of daily oral and LAI PrEP (i.e. “[Have you ever/in the last 6 months have you] taken PrEP (pre‐exposure prophylaxis) pills or had a PrEP injection to prevent HIV infection?”). Current daily oral PrEP users were coded as adherent users if they self‐reported taking ≥4 pills in the last week. LAI PrEP users were coded as adherent if they reported that it had been ≤9 weeks since last injection. Non‐adherence was defined as the complement (i.e. <4 pills or ≥9 weeks since last injection). Biomarkers of adherence were cost‐prohibitive given the sample size. LAI PrEP users were asked if they had used oral PrEP prior to LAI PrEP, the year of their first LAI injection, if they had ever received an injection late and if they had taken oral PrEP while waiting to receive a late injection.

Socio‐demographic characteristics were self‐reported and included age, race and ethnicity, location of residence, U.S. citizenship, education and food insecurity. Age was confirmed using the date of birth at study enrolment. Race and ethnicity were asked in two separate questions based on U.S. Census standards [16]. The race question used a “select all that apply” format. Response options included “White,” “Black or African American,” “Asian,” “American Indian or Alaskan Native,” “Hawaiian Native or Pacific Islander” or “other.” To assess ethnicity, participants were asked “Do you consider yourself to be Hispanic or Latina/o/x?”. All participants who selected “Black or African American” race, including multiracial participants and Afro‐Latina/o/x participants, were classified as Black race in regression models. All participants, regardless of race, who indicated that they identified as Hispanic or Latina/o/x were classified as Hispanic/Latina ethnicity in regression models. Zip code of residence was used to determine the corresponding U.S. Census Region and rurality. Participants were asked if they were U.S. citizens with three possible response options (“No,” “Yes, by birth” and “Yes, I became a citizen”). Educational attainment was dichotomized as high school diploma or less versus some college or higher. To assess for food security, participants were asked “In the past 6 months, was there a time when you/your household was worried whether food would run out before you would get money to buy more?”. Participants who responded “yes” were coded as food insecure.

Sexual health history included engagement in sex work, self‐reported recent STI diagnosis and perceived HIV risk. Participants who reported income from “sex work, survival sex, or sexual online service (such as Only Fans)” or reported that ≥1 partner in the last 6 months “gave money or other items of value in exchange for anal or vaginal sex” were considered to have engaged in sex work or exchanged sex in the prior 6 months. Participants who responded “yes” to “Have you tested positive for a sexually transmitted infection (STI) such as chlamydia or gonorrhoea in the last 6 months?” were coded as having tested positive for an STI. Participants were asked to self‐assess their HIV risk with the following question: “How high do you think your risk for HIV infection is?”. Response options included “No risk,” “Low,” “Medium” and “High.”

Healthcare access and utilization characteristics included health insurance (private, public or uninsured), receiving care at a clinic that specializes in LGBTQ, queer and/or trans health (LGBTQ+ health clinic; yes/no), receiving HIV/STI prevention information tailored for trans people (yes/no) and use of exogenous oestrogen and/or anti‐androgens in prior 6 months (yes/no). Additional correlates included gender pride (measured using the validated pride subscale of the Gender Minority Stress and Resilience Measure), and symptoms of psychological distress (measured using the Kessler‐6; dichotomized at a validated cut‐point where scores ≥13 indicate serious psychological distress) [17, 18].

### 2.3 Statistical analysis

The analytic sample was restricted to ENCORE participants who already use PrEP or may potentially benefit from PrEP. We broadly defined individuals who may benefit from PrEP as any individual who reported being sexually active and/or using a shared needle to inject drugs and/or silicone fillers in the prior 6 months. We characterized the HIVPC using descriptive statistics. For each step in the continuum, we assessed for differences in proportions between LAI and daily oral PrEP users using Fisher's exact test. Individuals who reported use of both LAI and oral PrEP in the prior 6 months were classified as LAI PrEP users for these comparisons. We descriptively summarized the socio‐demographic characteristics and experiences of individuals who reported using LAI PrEP. We used modified Poisson regression models with robust standard errors to estimate adjusted prevalence ratios (aPR) and 95% confidence intervals (95% CI) for correlates of uptake (among those who were PrEP‐aware) and non‐adherence (among current PrEP users). Variables that were significant at *p*<0.05 in bivariate regression models were retained in multivariable models, which also included potential confounders of age, race, ethnicity and U.S. Census Division. From the candidate correlates identified using expert opinion and prior studies, we conducted backward selection, removing one predictor at a time from the model until we ended up with a parsimonious model that minimized the Bayesian Information Criteria (BIC). As missingness was minimal (<5% across all variables), we used a complete case analysis to drop any observations that had at least one missing value.

## 3 RESULTS

### 3.1 Participant characteristics

Among participants enrolled in the ENCORE cohort from April 2023 to December 2024 (*N* = 2504), 1636 (65%) were individuals who already used PrEP or may benefit from PrEP and were included in our analysis. Among the study population of those who may benefit from PrEP, 42% were 18–29 years old, 18% identified as Hispanic and/or Latina/e/x and 13% identified as Black (inclusive of multiracial participants). Participants were distributed across the four U.S. Census regions (18% Northeast, 22% Midwest, 33% South and 27% West) and 10% resided in a Census‐designated rural area. The vast majority (77%) had attended some college or higher, and half (50%) experienced food insecurity in the prior 6 months. Additional socio‐demographic and healthcare access characteristics are reported in Table 1.

**Table 1:** Baseline characteristics of analytic sample of trans women vulnerable to HIV in the United States and Puerto Rico in 2023–2024 (*N* = 1636)

Age (years), median (interquartile range)	31 (26−39)
Age group	
18–29	687 (42.0%)
30–39	567 (34.7%)
40–49	235 (14.4%)
50+	147 (9.0%)
Race/ethnicity	
Non‐Hispanic White	991 (61.0%)
Non‐Hispanic Black	139 (8.6%)
Hispanic White	104 (6.4%)
Hispanic Black	25 (1.5%)
Non‐Hispanic and more than one race or other race	197 (12.1%)
Hispanic and more than one race or other race	169 (10.4%)
Race	
Black (inclusive of multiracial)	219 (13.4%)
Another race	1417 (86.6%)
Ethnicity	
Hispanic or Latina/e/x (of any race)	288 (17.7%)
Not Hispanic or Latina/e/x (of any race)	1338 (82.3%)
U.S. citizenship	
No	64 (3.9%)
Yes, by birth	1524 (93.7%)
Yes, by naturalization	39 (2.4%)
U.S. Census region	
Northeast	289 (17.7%)
Midwest	367 (22.4%)
South	546 (33.4%)
West	434 (26.5%)
Rural ZIP code	
No	1469 (90.3%)
Yes	158 (9.7%)
Education	
High school diploma/GED or less	370 (22.8%)
Some college of higher	1250 (77.2%)
Food security	
Food secure	799 (49.9%)
Food insecure	802 (50.1%)
Health insurance	
Uninsured	223 (13.8%)
Public insurance	598 (37.1%)
Private insurance	793 (49.1%)
Tested positive for STI in the last 6 months	
No	1516 (93.6%)
Yes	103 (6.4%)
Sex work or exchanged sex in the prior 6 months	
No	1146 (70.8%)
Yes	472 (29.2%)
Perceived risk for HIV acquisition	
No risk	275 (16.9%)
Low	816 (50.2%)
Medium	384 (23.6%)
High	150 (9.2%)
Receives care at LGBTQ+ health clinic	
No	729 (46.2%)
Yes	850 (53.8%)
Received HIV/STI prevention information tailored for trans people	
No	961 (59.0%)
Yes	559 (34.3%)
Don't know	110 (6.8%)
Used exogenous oestrogen and/or anti‐androgens in the last 6 months	
No	347 (21.2%)
Yes	1286 (78.8%)
Gender pride (16‐point continuous scale) (median, IQR)	12 (8−15)
Psychological distress	
None to moderate (Kessler‐6 score of 0–12)	948 (58.7%)
Serious (Kessler‐6 score of 13–24)	667 (41.3%)

### 3.2 HIV prevention continuum

**Figure 1:**
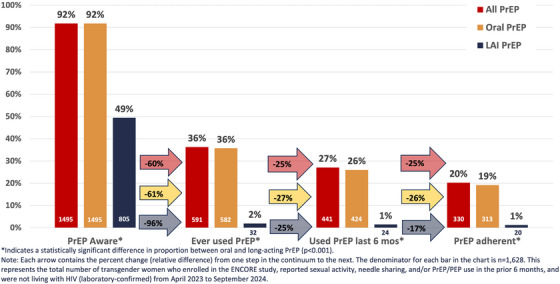
HIV prevention continuum among trans women not living with HIV who were sexually active or had a PrEP indication in the United States and Puerto Rico in 2023–2024 (*N* = 1628).

Participants had high levels of PrEP awareness, with 92% (1495/1628) reporting they had heard of PrEP (Figure 1). Eight participants declined to indicate whether they had heard of PrEP and were, therefore, excluded from further analysis. LAI PrEP awareness was significantly lower at 49% (805/1628) compared to 92% for daily oral (1495/1628; *p*<0.001). More than one‐third of the sample had ever used any form of PrEP (36%, 591/1628), and uptake was significantly lower for LAI PrEP at 2% (32/1628) compared to daily oral (36%, 582/1628; *p*<0.001). More than one quarter had used PrEP in the prior 6 months (27%, 441/1628), and the proportion was significantly higher for daily oral compared to LAI (26%, 424/1628 vs. 2%, 24/1628; *p*<0.001). One in five participants reported adherent use of PrEP (20%, 330/1628), including adherent users of daily oral (19%, 313/1628) and LAI (1%, 20/1628). The largest relative difference from one step in the continuum to the next was from awareness to uptake, with 60% of PrEP‐aware participants reporting they had never used PrEP (96% for LAI and 61% for daily oral). The majority of ever PrEP users had used PrEP in the last 6 months (75%; 73% for daily oral and 75% for LAI), and most recent PrEP users were adherent (75%; 74% for daily oral and 83% for LAI).

### 3.3 Characteristics of participants who have used LAI PrEP

Among the 32 participants who had ever used LAI PrEP, the median age was 32 years (range: 21–59), 19% (6/32) identified as Black and 34% (11/32) identified as Hispanic and/or Latina/x. Among 24 participants who used LAI PrEP in the prior 6 months, 79% (19/24) reported they had used daily oral PrEP prior to using LAI. Year of first LAI PrEP injection ranged from 2018 to 2024: 13% (3/24) first accessed LAI PrEP in 2018 through HPTN 083; 4% (1/24) started in 2022; 63% (15/24) started in 2023; and 21% (5/25) started in 2024. Of current LAI users, 17% (4/24) reported ever receiving an injection late, including 13% (3/24) who said there had been more than 13 weeks between injections (i.e. received an injection more than 4 weeks late). Of the four individuals who received a late injection, one reported using oral PrEP to cover the LAI interruption. It was not possible to determine adherence to oral PrEP during the interruption, so this participant was considered non‐adherent in additional analyses.

### 3.4 Correlates of step in the HIVPC—awareness to uptake

The prevalence ratios (PR) for PrEP uptake among PrEP‐aware participants are reported in Table 2. Factors positively associated with PrEP uptake in bivariate models included older age, Black race, Hispanic or Latina/e/x ethnicity, having public health insurance (compared to private), recent STI diagnosis (prior 6 months), sex work, greater perceived HIV risk, receiving healthcare at a LGBTQ+ clinic, having received information on HIV/STI that was specifically tailored for trans people, recent use of exogenous oestrogen and/or anti‐androgens, and higher levels of gender pride. Residing in the Midwest (compared to the West), having U.S. citizenship by birth and rural residence were inversely associated with PrEP uptake in bivariate models.

**Table 2:** Correlates of PrEP uptake among trans women in the United States and Puerto Rico who were aware of PrEP in 2023–2024 (*n* = 1495)

	PR	95% CI	*p*‐value	aPR	95% CI	*p*‐value
Age (years)	1.01	(1.01, 1.02)	<0.001	1.01	(1.01, 1.02)	<0.001
Black race (inclusive of multiracial; ref: another race)	1.31	(1.12, 1.54)	0.001	1.20	(1.01, 1.42)	0.033
Hispanic or Latina/e/x ethnicity (ref: not Hispanic of Latina/e/x)	1.35	(1.17, 1.56)	<0.001	1.19	(1.02, 1.38)	0.024
Citizen (ref: yes, by birth)						
No	1.36	(1.05, 1.76)	0.019			
Yes, by naturalization	1.46	(1.08, 1.97)	0.013			
Census region (ref: West)						
Northeast	1.00	(0.83, 1.20)	0.966	0.95	(0.79, 1.14)	0.593
Midwest	0.81	(0.67, 0.98)	0.030	0.80	(0.67, 0.96)	0.018
South	0.89	(0.76, 1.05)	0.178	0.90	(0.76, 1.05)	0.185
Rural zip code (ref: urban zip code)	0.55	(0.40, 0.76)	<0.001	0.72	(0.53, 0.99)	0.043
Education—some college of higher (ref: high school diploma/GED or less)	1.12	(0.95, 1.32)	0.189	1.22	(1.03, 1.44)	0.024
Food insecure (ref: food secure)	1.07	(0.94, 1.21)	0.313			
Health insurance (ref: private insurance)						
Uninsured	0.87	(0.69, 1.08)	0.204			
Public insurance	1.22	(1.07, 1.40)	0.004			
Tested positive for STI in the last 6 months (ref: did not test positive for STI in the last 6 months)	1.87	(1.61, 2.17)	<0.001	1.41	(1.17, 1.70)	<0.001
Sex work or exchange sex in the prior 6 months (ref: neither in the last 6 months)	1.31	(1.15, 1.49)	<0.001			
Perceived HIV risk (ref: no risk)						
Low	1.43	(1.12, 1.82)	0.004	1.40	(1.10, 1.79)	0.006
Medium	1.85	(1.44, 2.38)	<0.001	1.75	(1.36, 2.25)	<0.001
High	2.30	(1.76, 2.99)	<0.001	2.08	(1.59, 2.72)	<0.001
Receives care at LGBTQ+ health clinic (ref: does not receive care at LGBTQ+ clinic)	1.67	(1.45, 1.92)	<0.001	1.34	(1.16, 1.55)	<0.001
Healthcare provider knowledge of health issues facing trans people (ref: very knowledgeable)						
Not knowledgeable	0.62	(0.51, 0.76)	<0.001			
Somewhat knowledgeable	0.78	(0.67, 0.90)	0.001			
Don't know	0.51	(0.36, 0.71)	<0.001			
Received HIV/STI prevention information tailored for trans people (ref: did not receive tailored info)						
Yes	1.65	(1.45, 1.87)	<0.001	1.20	(0.89, 1.61)	0.232
Don't know	1.03	(0.76, 1.39)	0.855	1.49	(1.30, 1.70)	<0.001
Used exogenous oestrogen and/or anti‐androgens in the last 6 months (ref: did not use in the last 6 months)	1.37	(1.14, 1.66)	0.001	1.47	(1.21, 1.79)	<0.001
Gender pride (16‐point continuous scale, ref: 1‐point lower)	1.03	(1.02, 1.05)	<0.001	1.02	(1.00, 1.03)	0.047
Serious psychological distress (Kessler‐6 score ≥13: ref: little to moderate distress, Kessler‐6 score 0–12)	0.99	(0.87, 1.13)	0.837			

*Note*: Poisson regression with robust variance was implemented to estimate prevalence ratios. In multivariable models, backward selection was used to remove one predictor at a time from the model to fit a parsimonious model that minimized the Bayesian Information Criteria (BIC). Final model was adjusted for all other variables with an aPR displayed in the table.

Abbreviations: aPR, adjusted prevalence ratio; PR, prevalence ratio.

In the final adjusted model, the correlates with the largest magnitude of association included high self‐perceived HIV risk (aPR = 2.08, 95% CI = 1.59−2.72; *p*<0.001; ref: no risk), having received information on HIV/STI that was specifically tailored for trans people (aPR = 1.49, 95% CI = 1.30−1.70; *p*<0.001), recent use of exogenous oestrogen and/or anti‐androgens (aPR = 1.47, 95% CI = 1.21−1.79; *p*<0.001), recent STI diagnosis (aPR = 1.41, 95% CI = 1.17−1.70; *p*<0.001) and rural residence (aPR = 0.72, 95% CI = 0.53−0.99; *p* = 0.043). Individuals who received care at an LGBTQ+ clinic also had a higher prevalence of PrEP uptake compared to those who received their care somewhere else (aPR = 1.34, 95% CI = 1.16−1.55; *p*<0.001). For each 1‐point increase in gender pride, participants had a 2% higher prevalence of PrEP uptake (aPR = 1.02, 95% CI = 1.00−1.03; *p* = 0.047).

### 3.5 Correlates of step in the HIVPC—non‐adherence among current users

The PRs for PrEP non‐adherence among participants who had used PrEP within the prior 6 months are reported in Table 3. Factors associated with PrEP non‐adherence in bivariate models included being a non‐U.S. citizen (ref: citizen by birth), food insecurity and having a provider perceived to not be knowledgeable about the health issues facing trans people (ref: provider very knowledgeable). The final multivariable model was adjusted for age, race, ethnicity and census region. Although not significant in the bivariate model, in the multivariable model, older age was marginally associated with lower prevalence of non‐adherence (aPR = 0.99, 95% CI = 0.97−1.00; *p* = 0.086). Variables associated with non‐adherence in the final adjusted model included being a non‐U.S. citizen (aPR = 2.41, 95% CI = 1.44−4.05; *p* = 0.001; ref: citizen by birth) and experiencing food insecurity (aPR = 1.47, 95% CI = 1.04−2.06; *p* = 0.028).

**Table 3:** Correlates of PrEP non‐adherence among trans women who used PrEP in prior 6 months in the United States and Puerto Rico in 2023–2024 (*n* = 441)

	PR	95% CI	*p*‐value	aPR	95% CI	*p*‐value
Age (years)	0.99	(0.98, 1.01)	0.214	0.99	(0.97, 1.00)	0.086
Black race (inclusive of multiracial; ref: another race)	0.94	(0.61, 1.47)	0.799	0.94	(0.59, 1.50)	0.795
Hispanic or Latina/e/x ethnicity (ref: not Hispanic of Latina/e/x)	0.82	(0.53, 1.26)	0.362	0.67	(0.41, 1.10)	0.111
Citizen (ref: yes, by birth)						
No	2.06	(1.29, 3.30)	0.003	2.41	(1.44, 4.05)	0.001
Yes, by naturalization	0.55	(0.15, 2.02)	0.368	0.62	(0.18, 2.19)	0.458
Census region (ref: West)						
Northeast	0.96	(0.58, 1.56)	0.858	1.02	(0.63, 1.63)	0.943
Midwest	1.02	(0.65, 1.60)	0.932	0.99	(0.62, 1.58)	0.955
South	0.91	(0.60, 1.39)	0.674	0.91	(0.59, 1.41)	0.676
Rural zip code (ref: urban zip code)	1.10	(0.55, 2.23)	0.788			
Education—some college of higher (ref: high school diploma/GED or less)	0.93	(0.60, 1.44)	0.759			
Food insecure (ref: food secure)	1.41	(1.01, 1.96)	0.044	1.47	(1.04, 2.06)	0.028
Health insurance (ref: private insurance)						
Uninsured	0.94	(0.48, 1.82)	0.846			
Public insurance	1.30	(0.93, 1.83)	0.127			
Tested positive for STI in the last 6 months (ref: did not test positive for STI in the last 6 months)	1.40	(0.93, 2.11)	0.107			
Sex work or exchange sex in prior 6 months (ref: neither in the last 6 months)	1.30	(0.94, 1.81)	0.114			
Perceived HIV risk (ref: no risk)						
Low	1.86	(0.86, 4.07)	0.117			
Medium	2.03	(0.92, 4.49)	0.080			
High	1.60	(0.67, 3.82)	0.285			
Receives care at LGBTQ+ health clinic (ref: does not receive care at LGBTQ+ clinic)	1.03	(0.72, 1.47)	0.884			
Healthcare provider knowledge of health issues facing trans people (ref: very knowledgeable)						
Not knowledgeable	1.61	(1.06, 2.45)	0.027			
Somewhat knowledgeable	1.09	(0.74, 1.60)	0.660			
Don't know	1.38	(0.64, 2.97)	0.406			
Received HIV/STI prevention information tailored for trans people (ref: did not receive tailored info)						
Yes	1.31	(0.93, 1.85)	0.119			
Don't know	0.92	(0.40, 2.10)	0.838			
Used exogenous oestrogen and/or anti‐androgens in the last 6 months (ref: did not use in the last 6 months)	0.93	(0.59, 1.46)	0.746			
Gender pride (16‐point continuous scale, ref: 1‐point lower)	0.99	(0.95, 1.03)	0.524			
Serious psychological distress (Kessler‐6 score ≥13: ref: little to moderate distress, Kessler‐6 score 0–12)	1.04	(0.74, 1.45)	0.830			

*Note*: Poisson regression with robust variance was implemented to estimate prevalence ratios. In multivariable models, backward selection was used to remove one predictor at a time from the model to fit a parsimonious model that minimized the Bayesian Information Criteria (BIC). Final model was adjusted for all other variables with an aPR displayed in the table.

Abbreviations: aPR, adjusted prevalence ratio; PR, prevalence ratio.

## 4 DISCUSSION

In this nationwide cohort of trans women in the United States and Puerto Rico, we found that an overwhelming majority of trans women who may benefit from PrEP were aware of PrEP, and yet only one in three had ever used PrEP. Among trans women who may have benefitted from PrEP, just one in five were adherent PrEP users. These findings are similar to those from prior studies that have assessed PrEP awareness and uptake among transgender women in metropolitan areas in the United States [9−12]. Further, despite the FDA approval of LAI PrEP in 2021, only 49% of trans women who could benefit from PrEP in this study were aware of LAI PrEP, and just 2% had used LAI PrEP—a majority of whom had used daily oral PrEP previously—suggesting that awareness of and uptake of LAI PrEP among trans women are still relatively limited. Finally, three in four current LAI PrEP users were adherent, a similar proportion to prior single‐site studies with trans women from New York City and Oregon [19, 20]. Taken together, these data underscore the persistent chasm between PrEP awareness and uptake among trans women, as well as the urgent need for interventions to support uptake of and adherence to the approved PrEP formulations.

We found that PrEP uptake was highly associated with perceived HIV risk, which aligns with the theorized relationship between perceived susceptibility and likelihood of engaging in health‐promoting behaviour put forth in the Health Belief Model [21]. Prior studies have also highlighted the importance of perceived HIV risk in PrEP uptake [10, 22]. Similarly, we found trans women who had received an STI diagnosis in the prior 6 months had a 40% higher prevalence of PrEP uptake compared to those who had not. Given that PrEP care typically entails quarterly clinical visits, it is possible that individuals using PrEP are tested for STIs more frequently than those not using PrEP, that STI diagnoses lead to conversations about PrEP with providers or that those on PrEP are having more condomless sex, increasing STI acquisition risk. Due to the cross‐sectional nature of these data, we are not able to determine temporality between PrEP uptake and STI diagnosis. Individuals who received information on HIV/STIs that was specifically tailored for trans people had 49% higher prevalence of PrEP uptake, and individuals who received care at a clinic that specializes in LGBTQ+ health had 34% higher prevalence of PrEP uptake, suggesting that access to this culturally tailored care and specialized support could be vital to improving PrEP uptake among trans women. Echoing other studies that have demonstrated the positive association between access to gender‐affirming care and uptake of other preventative health services [23], we also found that trans women who had used exogenous hormones and/or anti‐androgens in the prior 6 months had 47% higher prevalence of PrEP uptake. Finally, the finding that those living in rural areas had 28% lower prevalence of PrEP uptake is unsurprising, given the well‐documented barriers to healthcare that trans individuals living in rural areas face, which may indicate a need for telehealth interventions to address these barriers [24−26].

We also found that trans women who were not U.S. citizens were more likely to have non‐adherent PrEP use compared to trans women who were U.S. citizens by birth. This finding may be explained by higher levels of depressive symptoms, employment discrimination, unstable housing and fear of deportation that undocumented trans women face compared to their documented counterparts, which could lead to care interruptions and/or poor adherence to preventative health behaviours [3]. It is important to note that we were not able to distinguish non‐citizen legal permanent residents from undocumented individuals for the purposes of this analysis, although many of these disproportionate health outcomes also impact trans women who have documentation but are not U.S. citizens [27, 28]. Finally, we found that individuals with food insecurity had 47% higher prevalence of non‐adherence compared to individuals who were food secure, which is especially concerning given evidence of shared socio‐structural drivers of food insecurity and HIV acquisition among trans women in the United States [4].

### 4.1 Limitations

This study is subject to some important limitations. First, these data are self‐reported and, as such, misclassification due to social desirability bias is possible. Although prior research from PrEP clinical trials suggests that self‐reported adherence may overestimate adherence compared to pharmacologic measures, recent implementation studies have reported moderate to high correlation between self‐reported and pharmacologic adherence measures [29−32]. Nonetheless, we acknowledge the limitations of self‐reported adherence data. Second, although we had initially planned to assess for predictors of LAI uptake and to compare the socio‐demographics of LAI versus daily oral PrEP users, we had too few LAI PrEP users in our sample to do so. As LAI access expands and more trans women initiate LAI PrEP, additional research is needed to assess for these differences. Further, this analysis did not address differential access to PrEP based on urbanicity or other indicators associated with PrEP access, which may limit generalizability. Additionally, because our definition of current PrEP use was based on a survey item with a 6‐month recall, it is possible that some individuals we classified as non‐adherent current PrEP users had appropriately discontinued PrEP based on changes in risk; if true, we may have overestimated non‐adherence. Next, the concept of the HIVPC explicitly acknowledges the important role of efficacious HIV prevention strategies beyond PrEP; however, for simplicity, this analysis more narrowly focused on PrEP and did not capture engagement in other HIV prevention strategies. Therefore, it is possible that individuals in our sample were using other efficacious prevention strategies, which may not be accounted for. Additionally, our definition of individuals who may benefit from PrEP, which broadly included individuals who reported being sexually active and/or using a shared needle to inject drugs and/or silicone fillers in the prior 6 months, may have resulted in heterogeneity in HIV acquisition risk and likelihood of benefitting from PrEP. Although we have several procedures in place to prevent and identify fraudulent and duplicate enrolments, there is always some risk of fraudulent enrolment in studies reliant on remotely collected data. Finally, due to the cross‐sectional nature of these data, correlates of step in the HIVPC do not imply a causal relationship; additional longitudinal studies are needed to identify predictors of step in the HIVPC.

## 5 CONCLUSIONS

We found suboptimal PrEP uptake and adherence among trans women and that modifiable factors related to healthcare access and utilization (such as receiving care at an LGBTQ+ clinic) and other social determinants of health (such as citizenship status and food security) are associated with progression in the HIVPC. These data are among the first to describe the PrEP continuum among a national sample of trans women in the United States and Puerto Rico. Our findings demonstrate low levels of PrEP uptake, particularly for LAI PrEP. These data corroborate existing literature, which suggests that PrEP uptake has not been optimized among trans women in the United States and that interventions to improve PrEP outcomes, including uptake and adherence, are needed.

## COMPETING INTERESTS

EEC, ALW and TCP received funding support from ViiV healthcare to support this analysis through a grant to their institutions. VV, LR and AG are employed by ViiV Healthcare and have received stocks in GSK. CAB was employed by ViiV Healthcare and received stocks in GSK while this manuscript was in development. No other financial disclosure was reported.

## AUTHOR CONTRIBUTIONS

EEC, TCP, MS, AER, AB, KNA, SL, CP, AL, MM, CB, SLR and ALW implemented the research. EEC analysed the data, generated all figures and tables, and wrote the first draft. ALW and SLR acquired funding to support this research. All authors have provided substantive feedback, including input on the analytic approach and data interpretation. All authors have read, edited and approved the final manuscript. The following individuals are members of the collaborative authorship group, Enhanced Cohort Methods for HIV Research and Epidemiology Among Transgender Women in the United States (ENCORE) Study Group: University of Michigan: Sari Reisner (mPI); Johns Hopkins University: Andrea Wirtz (mPI), Keri Althoff, Erin Cooney, James Case, Elizabeth Humes, **Sabriya Linton**, **Ceza Pontes**, Megan Stevenson, Camille White; Arianna's Center: **Arianna Lint**; Black Transgender Advocacy Coalition: **Carter Brown**; Callen‐Lorde Community Health Center: Asa Radix; Duke University: Tonia Poteat, **Chris Beyrer**; Emory University and Grady Hospital: Jason Schneider, J. Sonya Haw; Trans Solutions: **Marissa Miller**; University of Miami: Andrew Wawrzyniak, Allan Rodriguez; University of California San Diego: **Annick Borquez**.

## FUNDING

Research reported in this publication is supported by the Eunice Kennedy Shriver National Institute Of Child Health & Human Development (NICHD), National Institute Of Allergy And Infectious Diseases (NIAID), National Institute On Alcohol Abuse And Alcoholism (NIAAA) of the National Institutes of Health under the award R01AI172092 (ALW and SLR). The Enhanced Cohort Methods for HIV Research and Epidemiology (ENCORE) study is appreciative of support from ViiV Healthcare and infrastructure support from the Centers for AIDS Research (CFAR) at partner institutions, including Johns Hopkins University (P30AI094189), Duke University (P30 AI064518), Emory University (P30AI050409) and the University of Miami (P30AI073961).

## DISCLAIMER

The content is solely the responsibility of the authors and does not necessarily represent the official views of the National Institutes of Health.

## ETHICS APPROVAL STATEMENT

The Johns Hopkins Single Institutional Review Board reviewed and approved this study (IRB00355445) and served as the institutional review board of record for all partner institutions in this multisite study. As a minimal risk study, participants were asked to provide consent in English or Spanish using an oral consent form in web‐based format prior to initiating research activities.

## Data Availability

Deidentified individual data and a data dictionary will be made available upon reasonable request after the signing of a data use agreement. There is a formal process for external users to request access to cohort data, which involves review and approval by principal investigators from each study site and the community advisory board. Further details and forms can be obtained by emailing ALW and SLR.

## References

[1] Becasen JS , Denard CL , Mullins MM , Higa DH , Sipe TA . Estimating the prevalence of HIV and sexual behaviors among the US transgender population: a systematic review and meta‐analysis, 2006–2017. Am J Public Health. 2019;109(1):e1–e8. 10.2105/AJPH.2018.304727 PMC6301428 30496000

[2] Baral SD , Poteat T , Stromdahl S , Wirtz AL , Guadamuz TE , Beyrer C . Worldwide burden of HIV in transgender women: a systematic review and meta‐analysis. Lancet Infect Dis. 2013;13(3):214–222. 23260128 10.1016/S1473-3099(12)70315-8

[3] Yamanis T , Malik M , Del Rio‐Gonzalez AM , Wirtz A , Cooney E , Lujan M , et al. Legal immigration status is associated with depressive symptoms among Latina transgender women in Washington, DC. Int J Environ Res Public Health. 2018;15(6):1246. 29895781 10.3390/ijerph15061246 PMC6025023

[4] Zubizarreta D , Wirtz AL , Humes E , Cooney EE , Stevenson M , Althoff KN , et al. Food insecurity is high in a multi‐site cohort of transgender women vulnerable to or living with HIV in the eastern and southern United States: baseline findings from the LITE cohort. Nutrients. 2024;16(5):707. 38474837 10.3390/nu16050707 PMC10933826

[5] Fletcher JB , Kisler KA , Reback CJ . Housing status and HIV risk behaviors among transgender women in Los Angeles. Arch Sex Behav. 2014;43(8):1651–1661. 25190499 10.1007/s10508-014-0368-1 PMC4214608

[6] Golub SA , Gamarel KE , Rendina HJ , Surace A , Lelutiu‐Weinberger CL . From efficacy to effectiveness: facilitators and barriers to PrEP acceptability and motivations for adherence among MSM and transgender women in New York City. AIDS Patient Care STDs. 2013;27(4):248–254. 23565928 10.1089/apc.2012.0419 PMC3624632

[7] Teng F , Sha Y , Fletcher LM , Welsch M , Burns P , Tang W . Barriers to uptake of PrEP across the continuum among transgender women: a global scoping review. Int J STD AIDS. 2023;34(5):299–314. 36793197 10.1177/09564624231152781

[8] McNairy ML , El‐Sadr WM . A paradigm shift: focus on the HIV prevention continuum. Clin Infect Dis. 2014;59(suppl_1):S12–S15. 24926026 10.1093/cid/ciu251 PMC4141493

[9] Poteat T , Wirtz A , Malik M , Cooney E , Cannon C , Hardy WD , et al. A gap between willingness and uptake: findings from mixed methods research on HIV prevention among Black and Latina transgender women. J Acquir Immune Defic Syndr. 2019;82(2):131–140. 31180995 10.1097/QAI.0000000000002112 PMC7807529

[10] Malone J . Perceived HIV acquisition risk and low uptake of PrEP among a cohort of transgender women with PrEP indication in the eastern and southern United States. J Acquir Immune Defic Syndr. 2021;88(1):10‐18. 34397742 10.1097/QAI.0000000000002726 PMC8371736

[11] Wilson EC , Turner CM , Arayasirikul S , Lightfoot M , Scheer S , Raymond HF , et al. Disparities in the PrEP continuum for trans women compared to MSM in San Francisco, California: results from population‐based cross‐sectional behavioural surveillance studies. J Int AIDS Soc. 2020;23(Suppl 3):e25539. 32602642 10.1002/jia2.25539 PMC7325513

[12] Morris E , Teplinskaya A , Olansky E , Rinderle JK , Chapin‐Bardales J , National HIVBSATWSG . Characteristics associated with pre‐exposure prophylaxis discussion and use among transgender women without HIV infection—National HIV Behavioral Surveillance among transgender women, seven urban areas, United States, 2019–2020. MMWR. 2024;73(Suppl 1):9–20. 10.15585/mmwr.su7301a2 PMC10826686 38261546

[13] Wirtz AL , Poteat T , Borquez A , Linton S , Stevenson M , Case J , et al. Enhanced Cohort Methods for HIV Research and Epidemiology (ENCORE): protocol for a nationwide hybrid cohort for transgender women in the United States. JMIR Res Protoc. 2024;13:e59846. 39190916 10.2196/59846 PMC11387927

[14] Fauci AS , Redfield RR , Sigounas G , Weahkee MD , Giroir BP . Ending the HIV epidemic: a plan for the United States. JAMA. 2019;321(9):844–845. 30730529 10.1001/jama.2019.1343

[15] Reisner SL , Humes E , Stevenson M , Cooney EE , Adams D , Althoff KN , et al. Site‐based and digital cohort participation among transgender women in the eastern and southern United States: findings from the LITE study. J Acquir Immune Defic Syndr. 2024;97(5):e10–e24. 39261981 10.1097/QAI.0000000000003527 PMC11987987

[16] Jones NA . Update on the US Census Bureau's race and ethnic research for the 2020 census. Survey News. 2017;3(5):3.

[17] Testa RJ , Habarth J , Peta J , Balsam K , Bockting W . Development of the gender minority stress and resilience measure. Psychol Sex Orient Gender Divers. 2015;2(1):65.

[18] Kessler RC , Andrews G , Colpe LJ , Hiripi E , Mroczek DK , Normand S‐LT , et al. Short screening scales to monitor population prevalences and trends in non‐specific psychological distress. Psychol Med. 2002;32(6):959–76. 12214795 10.1017/s0033291702006074

[19] Starbuck L , Golub SA , Klein A , Harris AB , Guerra A , Rincon C , et al. Brief Report: transgender women and preexposure prophylaxis care: high preexposure prophylaxis adherence in a real‐world health care setting in New York City. J Acquir Immune Defic Syndr. 2022;90(1):15–9. 35013087 10.1097/QAI.0000000000002915 PMC8986585

[20] Downing J , Yee K , Sevelius JM . PrEP use and adherence among transgender patients. AIDS Behav. 2022;26(4):1251–1259. 34643827 10.1007/s10461-021-03482-8 PMC9351441

[21] Rosenstock IM , Strecher VJ , Becker MH . Social learning theory and the Health Belief Model. Health Educ Q. 1988;15(2):175–183. 3378902 10.1177/109019818801500203

[22] Felsher M , Szep Z , Krakower D , Martinez‐Donate A , Tran N , Roth AM . “I don't need PrEP right now”: a qualitative exploration of the barriers to PrEP care engagement through the application of the health belief model. AIDS Educ Prev. 2018;30(5):369–381. 30332306 10.1521/aeap.2018.30.5.369 PMC8558876

[23] Restar AJ . Gender‐affirming care is preventative care. Lancet Reg Health Am. 2023;24:100544. 37383047 10.1016/j.lana.2023.100544 PMC10290445

[24] Knutson D , Koch JM , Arthur T , Mitchell TA , Martyr MA . “Trans broken arm”: health care stories from transgender people in rural areas. 2016. 10.1089/trgh.2017.0052 PMC6004081 29915810

[25] Renner J , Blaszcyk W , Täuber L , Dekker A , Briken P , Nieder TO . Barriers to accessing health care in rural regions by transgender, non‐binary, and gender diverse people: a case‐based scoping review. Front Endocrinol. 2021;12:717821. 10.3389/fendo.2021.717821 PMC8637736 34867775

[26] Tillewein H , Becker J , Kruse‐Diehr A . Institutional barriers to healthcare services among transgender individuals in the rural Midwest. J Homosex. 2024;71(9):2099–2115. 37289135 10.1080/00918369.2023.2222204

[27] Gonzalez KA , Abreu RL , Rosario CC , Koech JM , Lockett GM , Lindley L . “A center for trans women where they help you”: resource needs of the immigrant Latinx transgender community. Int J Transgend Health. 2022;23(1–2):60–78. 35403119 10.1080/26895269.2020.1830222 PMC8986257

[28] Palazzolo SL , Yamanis TJ , De Jesus M , Maguire‐Marshall M , Barker SL . Documentation status as a contextual determinant of HIV risk among young transgender Latinas. LGBT Health. 2016;3(2):132–138. 26669583 10.1089/lgbt.2015.0133 PMC4841909

[29] Qasmieh S , Nash D , Gandhi M , Rozen E , Okochi H , Goldstein H , et al. Self‐reported use of HIV preexposure prophylaxis is highly accurate among sexual health clinic patients in New York City. Sex Transm Dis. 2022;49(11):790–793. 35312670 10.1097/OLQ.0000000000001622 PMC9463403

[30] Landovitz RJ , Beymer M , Kofron R , Amico KR , Psaros C , Bushman L , et al. Plasma tenofovir levels to support adherence to TDF/FTC preexposure prophylaxis for HIV prevention in MSM in Los Angeles, California. J Acquir Immune Defic Syndr. 2017;76(5):501–511. 28902074 10.1097/QAI.0000000000001538 PMC5681370

[31] Montgomery MC , Oldenburg CE , Nunn AS , Mena L , Anderson P , Liegler T , et al. Adherence to pre‐exposure prophylaxis for HIV prevention in a clinical setting. PLoS One. 2016;11(6):e0157742. 27333000 10.1371/journal.pone.0157742 PMC4917105

[32] Tan DHS , Schnubb A , Lawless J , Szadkowski L , Grennan T , Wilton J , et al. Acceptability and tolerability of and adherence to HIV preexposure prophylaxis among Toronto gay and bisexual men: a pilot study. CMAJ Open. 2018;6(4):E611–E617. 10.9778/cmajo.20180068 PMC6287974 30530721

